# Multi-Omic Analysis Reveals the Molecular Mechanism of UV-B Stress Resistance in Acetylated *RcMYB44* in *Rhododendron chrysanthum*

**DOI:** 10.3390/genes14112022

**Published:** 2023-10-30

**Authors:** Meiqi Liu, Xiaoru Lin, Kun Cao, Liping Yang, Hongwei Xu, Xiaofu Zhou

**Affiliations:** Jilin Provincial Key Laboratory of Plant Resource Science and Green Production, Jilin Normal University, Siping 136000, Chinaxuhongwei@jlnu.edu.cn (H.X.)

**Keywords:** *R. chrysanthum*, UV-B radiation, transcriptome, acetylated proteome, hormone signal transduction, *RcMYB44*

## Abstract

Ultraviolet-B (UV-B) radiation is a significant environmental factor influencing the growth and development of plants. MYBs play an essential role in the processes of plant responses to abiotic stresses. In the last few years, the development of transcriptome and acetylated proteome technologies have resulted in further and more reliable data for understanding the UV-B response mechanism in plants. In this research, the transcriptome and acetylated proteome were used to analyze *Rhododendron chrysanthum* Pall. (*R. chrysanthum*) leaves under UV-B stress. In total, 2348 differentially expressed genes (DEGs) and 685 differentially expressed acetylated proteins (DAPs) were found. The transcriptome analysis revealed 232 MYB TFs; we analyzed the transcriptome together with the acetylated proteome, and screened 4 MYB TFs. Among them, only *RcMYB44* had a complete MYB structural domain. To investigate the role of RcMYB44 under UV-B stress, a homology tree was constructed between *RcMYB44* and *Arabidopsis* MYBs, and it was determined that *RcMYB44* shares the same function with *ATMYB44*. We further constructed the hormone signaling pathway involved in *RcMYB44*, revealing the molecular mechanism of resistance to UV-B stress in *R. chrysanthum*. Finally, by comparing the transcriptome and the proteome, it was found that the expression levels of proteins and genes were inconsistent, which is related to post-translational modifications of proteins. In conclusion, *RcMYB44* of *R. chrysanthum* is involved in mediating the growth hormone, salicylic acid, jasmonic acid, and abscisic acid signaling pathways to resist UV-B stress.

## 1. Introduction

Abiotic stresses encountered by a plant at every moment of its life can severely affect its growth and development. A plant’s genes, or portions of them, cooperate with each other to regulate growth and developmental processes and to help in defence against adverse environments [1,2]. So, finding the vital genes or proteins participating in the plant’s response to abiotic stresses and inquiring into the molecular mechanisms of stress resistance can lay a solid foundation for breeding varieties that are resistant to abiotic stresses. Transcription factors have at least two structural domains: the DNA-binding domain and the transcriptional activation domain. Transcription factors play a critical role in plant growth and development by activating or repressing the physiological and biochemical processes of transcription in response to exogenous or endogenous stimuli when the plant is in an unfavorable environment [3]. In fact, the MYBs play an essential role in the processes of plant responses to abiotic stresses.

Researches have reported that the MYB gene family plays an essential role in plants’ responses to abiotic stresses. In wheat, several MYBs responsive to stress have been discovered, including *TaMYB1*, which is capable of a range of responses when wheat is subjected to abiotic stress, and ABA, *TaMYB2A*, which exhibits tolerance to multiple abiotic stresses in transgenic *Arabidopsis thaliana*, and *TaMYB33*, which re-establishes osmotic homeostasis and enhances salinity and drought tolerance in transgenic plants [4,5,6]. *Arabidopsis thaliana*, as a model organism, has also been reported to have many MYBs involved in the abiotic stress response. For example, *AtMYB52* overexpression makes plants drought tolerant and regulates cell wall biosynthesis. *AtMYB33*, *AtMYB65*, and *AtMYB101*, which can replace barley (*Hordeum Uggare*) GAMYB, transactivate the barley α-amylase promoter and mediate GA signaling effects during flowering. Similarly, *AtMYB44*, *AtMYB77*, *AtMYB73*, and *AtMYB70* have important functions in plant responses to abiotic stresses.

*R. chrysanthum* is distributed in the Alpine tundra zone of Changbai Mountain, where the plant is exposed to abiotic stresses such as UV-B radiation. Its unique growing environment endows it with resistance to UV-B stress, so, *R. chrysanthum* is a good plant material for the study of abiotic stresses [7,8]. UV-B (ultraviolet-B) radiation is a type of ultraviolet radiation with wavelengths between 280 and 320 nm. Normally, UV-B radiation is mostly absorbed by the ozone layer, and only a small amount of UV-B radiation reaches the Earth’s surface [8]. However, today’s highly polluted atmosphere and the destruction of the ozone layer have resulted in an excess of UV-B light reaching the Earth’s surface [9,10,11]. Plants, as producers in the ecosystem, have a clear response to UV-B radiation [12]. Excessive UV-B radiation has been shown to have severe effects on plants in terms of their morphology, physiology, biochemistry, and cellular activity [13,14,15,16]. Therefore, we selected *R. chrysanthum* as the experimental material to explore the molecular mechanism of plant resistance to UV-B radiation.

In a previous study, we irradiated wild-type and domesticated *R. chrysanthum* with PAR, PAR + UV-A, and PAR + UV-B for 2 days. The results showed that UV-B radiation inhibited photosynthesis in *R. chrysanthum*, while wild-type *R. chrysanthum* activated its resistance to UV-B stress [17]. Subsequently, we investigated the response of antioxidant enzymes to abiotic stresses using a proteomic approach, revealing their important role in the resistance to stress [7]. A metabolomic analysis showed that the expression of flavonoids, organic acids, amino acids, and fatty acids was up-regulated in plants under UV-B stress, making them resistant to UV-B radiation, but transcriptome analysis showed consistent changes in the content of genes and metabolites related to sucrose and starch metabolism, and the opposite for amino acid metabolism [18]. This is because the mechanisms of resistance in plants are extremely complex. They may be related to the expression of proteins, and some proteins will undergo post-translational modification (PTM), which will affect the expression of proteins, and then affect the expression of metabolites. It is, therefore, extremely important to continue to unravel the mechanism of the resistance of plants to UV-B radiation.

Lysine acetylation has been found to be an abundant and important PTM that affects a variety of important biological processes in organisms [19,20,21]. Indeed, PTMs play a vital role in several signaling pathways in plants, and they mainly harmonize protein functions [22] by altering the activities, subcellular localization, and stability of proteins [23]. Lysine acetylation (Kac) has been extensively studied and found to exhibit a prominent role during plant resistance to abiotic stress [24,25,26,27,28]. In the last few years, developments in proteomics have provided a foundation for understanding the lysine acetylation proteome and new directions for investigating the extent and regulatory mechanisms of the non-histone protein Kac [29]. However, although the Kac contents of *Arabidopsis thaliana* [30,31], poplar (*Populus tremula × Populus alba*) [32], rice (*Oryza sativa*) [33,34], pea (*Pisum sativum* L.) [35], and wheat (*Triticum Aestivum*) have been intensively investigated [36], there have been very few studies on the Kac content of *R. chrysanthum*.

In this work, the transcriptome and acetylated proteome are analyzed to investigate *RcMYB44*, which undergoes acetylation modification under UV-B stress, and its functions, and to reveal the molecular mechanism of the resistance of the *RcMYB44* gene of *R. chrysanthum* to UV-B stress.

## 2. Materials and Methods

### 2.1. Plant Material

*R. chrysanthum* plants were kept in an artificial climate chamber. The plants were grown under white fluorescent lamps at 50 μmol (photons) m^−2^ s^−1^. The chamber was set to 14 h of light at 18 °C and 10 h of darkness at 16 °C, with a relative humidity of 60%. 

### 2.2. Experimental Design

In order to investigate the molecular mechanism of UV-B resistance in *R. chrysanthum*, the *R. chrysanthum* plants were divided into two groups of three replicates each. One group was placed in photosynthetically active radiation (PAR) for 48 h (CG) and the other group was placed in PAR and UV-B radiation for 48 h (BG).

### 2.3. PAR and UV-B Radiation Exposure

The plants were placed under artificial radiation, UV-B (280–315 nm) and PAR (400–700 nm), replicated for each group (n = 3). In order to obtain the two radiation environments, different filters, with different transmissions, were used. In the PAR treatment, a 400 nm long-pass filter (Edmund, Filter Long 2IN SQ, Barrington, NJ, USA) was placed above the vials of the plants. In the PAR + UV-B treatments, a 295 nm long-pass filter (Edmund, Filter Long 2IN SQ, Barrington, NJ, USA) was used. In the experiments, visible light (PAR) was provided by warm-white fluorescent lamps (Philips, T5 × 14 W, Amsterdam, The Netherlands). UV-B fluorescence tubes (Philips, Ultraviolet-B TL 20 W/01 RS, Amsterdam, The Netherlands) were used as artificial UV-B radiation sources. The samples effectively received an irradiance of 2.3 W m^−2^ of UV-B with a PAR of 50 µmol (photon) m^−2^ s^−1^ based on the transmission function of the long-pass filter.

### 2.4. Transcriptomics Analysis

The whole RNA was treated using either rRNA removal or mRNA enrichment. With the use of magnetic beads and OligodT, mRNA with polyA tails was enhanced. To remove rRNA, rRNA was hybridized with a DNA probe, the DNA/RNA hybrid strand was selectively destroyed by RNaseH, the DNA probe was subsequently digested off using DNaseI, and the resulting product was purified. The obtained RNA was fragmented using an interrupted buffer, reverse-transcribed using a random N6 primer, and then the synthesized double-stranded DNA was flattened and phosphorylated at the 5′ end. Finally, a sticky end with an A protruded from the 3′ end of the double-stranded DNA. Then, a sticky end was joined to a 3′ end. In order to generate a sticky end with a protruding “A” at the 3′ end, and a bulge-like junction with a protruding “T” at the 3′ end, the ends of the synthesized double-stranded DNA were flattened and phosphorylated at the 5′ end. To create a single-stranded circular DNA library, the ligated product was amplified using PCR using particular primers; the PCR product was heat-denatured to single-stranded DNA, and finally, the single-stranded DNA was cyclized with a bridge primer. 

#### 2.4.1. Screening of Differentially Expressed Genes

The transcriptomics in this experiment was performed by Shenzhen Huada Gene Technology Research Co., Ltd. (Shenzhen, China), screening for differentially expressed genes. The *p*-values were calculated according to a normal distribution. The *p*-values were corrected to Q-values. To increase the precision, genes with more than double difference and Q-values ≤ 0.001 were selected as having significant differential expression.

#### 2.4.2. KEGG Enrichment Analysis of DEGs

We used the KEGG database for the enrichment analysis of the KEGG pathway. The R 4.0.5 software’s phyper function was used to calculate the *p*-values for the enrichment studies, which were then FDR-corrected. A Q-value < 0.05 is typically regarded as significantly enriched.

### 2.5. 4D Label-Free Quantitative Acetylated Proteomic Analysis 

**Figure 1:**
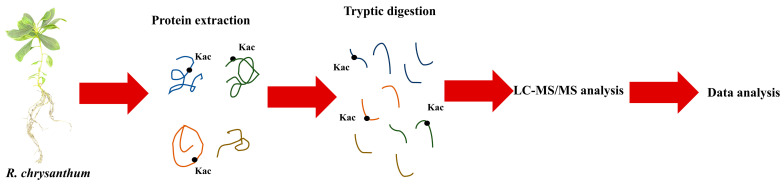
Experimental flow of acetylated proteome analysis.

The acetylated proteomics in this experiment was performed by Jingjie PTM Biolab using a 4D label-free analysis. The specific experimental technique flow is shown in Figure 1.

#### 2.5.1. Protein Extraction

Using a high-intensity ultrasound processor (Scientz, Ningbo, China), *R. chrysanthum* were crushed and pulverized in liquid nitrogen, transferred to centrifuge tubes, then treated three times on ice in a lysis buffer (8 M urea, 2 mM EDTA, 10 mM DTT, and 1% protease inhibitor cocktail). The supernatant was centrifuged for three minutes at 4 °C after being centrifuged at 20,000× *g* for ten minutes. The supernatant was centrifuged for 3 min at 4 °C to separate the proteins, and it was then precipitated with 15% TCA for 4 h at −20 °C. After chilling in a refrigerator, the remaining sediment was cleaned three times with acetone. Finally, the proteins were redissolved in a buffer consisting of 8 M urea and 100 mM TEAB, which had a pH of 8.0. The protein concentrations were estimated using a 2D quantitative analysis kit.

#### 2.5.2. Trypsin Digestion

TCA, at a concentration of 20% (*m*/*v*), was slowly added to the sample to precipitate the proteins, followed by vortex mixing and incubation at 4 °C for 2 h. The precipitated proteins were collected by centrifugation at 4500× *g* for 5 min at 4 °C. Subsequently, the collected precipitated proteins were washed with pre-cooled acetone three times and allowed to dry for 1 min. The washed and dried proteins were then redissolved in 200 mM TEAB and dispersed using ultrasonication. For the first digestion, trypsin was added at a 1:50 trypsin mass ratio overnight. Subsequently, the peptides were reduced with 5 mM dithiothreitol for 60 min at 37 °C, followed by 11 mM iodoacetamide for 45 min at room temperature and in the dark. Finally, the peptides were desalted using a Strata X SPE column.

#### 2.5.3. Affinity Enrichment

Tryptic peptides were dissolved in NETN buffer (100 mM NaCl, 1 mM EDTA, 50 mM Tris-HCl, 0.5% NP-40, pH 8.0) to dissolve Kac-modified peptides. Pre-washed antibody beads (PTM-104, PTM Bio, Hangzhou, China) were then added and incubated with the peptide mixture at 4 °C overnight with gentle shaking. The beads were then washed twice with water and four times with NETN buffer. Use of 0.1% trifluoroacetic acid allowed the bound peptides to be released from the beads. The eluted portions were then mixed and dried under vacuum. The resultant peptides were desalted using C18 ZipTips (Millipore, Burlington, ON, Canada), in accordance with the manufacturer’s instructions, for LC-MS/MS analysis.

#### 2.5.4. LC-MS/MS Analysis and Database Search

Solvent A (0.1% formic acid, 2% acetonitrile in water) was used to solubilize the tryptic peptides, which were then loaded directly onto a home-made analytical column (25 cm length, 100 μm i.d.). In a nanoElute UHPLC system (Bruker Daltonics, Billerica, MA, USA) running at 350 nL/min, the solvent B gradient was increased from 6% to 22% (0.1% formic acid dissolved in acetonitrile) over 42 min, increased from 22% to 30% in 12 min, increased to 80% in 3 min, maintained at 80%, and then held at 80% for 3 min. Prior to mass spectrometry analysis with a timsTOF Pro (Bruker Dalton, Billerica, MA, USA), the peptides were subjected to capillary source treatment. A 1.75 kV electrospray voltage was used. At the TOF detector, the precursors and fragments were analyzed using MS/MS with a scan range of 100 to 1700 *m*/*z*. Parallel accumulation serial fragmentation (PASEF) mode was used to run the timsTOF Pro. Ten PASEF-MS/MS scans were acquired per acquisition cycle, with the fragmentation of precursors with charge states of 0 to 5 being the preferred range. A 24 s dynamic exclusion was set. The MaxQuant search engine (v. 1.6.6.0) was used to process the generated MS/MS data. Up to 4 missing cleavage events were made possible by using the cleavage protein trypsin/P. The mass tolerance for the precursor ions was set to 20 ppm in both the first search and the main search. And the mass tolerance for the fragment ions was set as 20 ppm. Protein N-terminal acetylation, oxidation on Met, and acetylation on Lys were designated as variable modifications, while carbamidomethyl on Cys was designated as a fixed modification. The FDR was adjusted to <1%.

### 2.6. Bioinformatics Analysis

After averaging the protein expression in the CG and BG groups separately, the ratio of the averages of the CG and BG groups was calculated. This ratio was used for the quantitative results. The *p*-values were computed from two-tailed Student’s *t*-tests and log-transformed quantitative values. Values for proteins or modified peptides of *p* < 0.05 and a fold change (FC) > 1.5 were used to determine significant differences. 

### 2.7. Protein Homology Modeling

In order to understand the protein structure, NCBI was used to compare homologous sequences and to model the three-dimensional structure of proteins, and for the hydrophobic structural model and salt bridge model of the acetylated proteins.

### 2.8. Statistics and Analysis of Data

The protein data of the hormone signal transduction pathway in the CG group and BG group were analyzed, and the significance was analyzed using the SPSS Statistics 26 software.

## 3. Results

### 3.1. Acetylated Proteome and Transcriptome Comprehensive Analysis of Functional Classification and KEGG Enrichment in R. chrysanthum 

Under the PAR and UV-B treatments, 2348 DEGs were found. Among them, 1157 DEGs were increased and 1191 DEGs were decreased after UV-B stress (Table S1). A total of 807 proteins and 685 acetylated proteins were discovered from the leaves under the PAR and UV-B treatments (Tables S2 and S3). Among them, 450 DEPs were increased and 357 DEPs decreased after UV-B stress. After UV-B stress, 95 acetylated proteins and 104 sites were increased and 590 acetylated proteins and 841 sites were decreased. GO annotations on the DEGs showed that the sets of data were classified; among them were the “catalytic activity” term (755 DEGs) in molecular function and the “cellular process” term (486 DEGs) in biological process (Table S4). These results suggest that the response of *R. chrysanthum* to UV-B stress was related to processes such as cellular processes and catalytic activity. Remarkably, many of the DEGs participated in several vital processes of classifications that would affect UV-B tolerance in plants, for example, “antioxidant activity”, “transcription regulator activity”, and “response to stimulus”. Furthermore, the GO analysis also showed that a range of important biological processes, cellular components, and molecular functions differed in UV-B-stressed *R. chrysanthum*. Overall, these data offer valuable insights for an understanding of the molecular mechanisms of *R. chrysanthum* responses to UV-B stress. GO annotations of the DEPs and DAPs showed that the sets of data were classified. Among them, the “cell” term (461 DEPs and 427 DAPs) in cellular component, “cellular process” term (298 DEPs and 295 DAPs) in biological process, and “catalytic activity” term (199 DEPs and 255 DAPs) in molecular function (Table S5). These results indicate that the response of *R. chrysanthum* to UV-B stress focuses on cells, cellular processes, and catalytic activity. Several important biological processes, molecular functions, and cellular components were different in *R. chrysanthum* during UV-B exposure.

### 3.2. Prediction and Analysis of RcMYB44 Function under UV-B Stress

**Figure 2:**
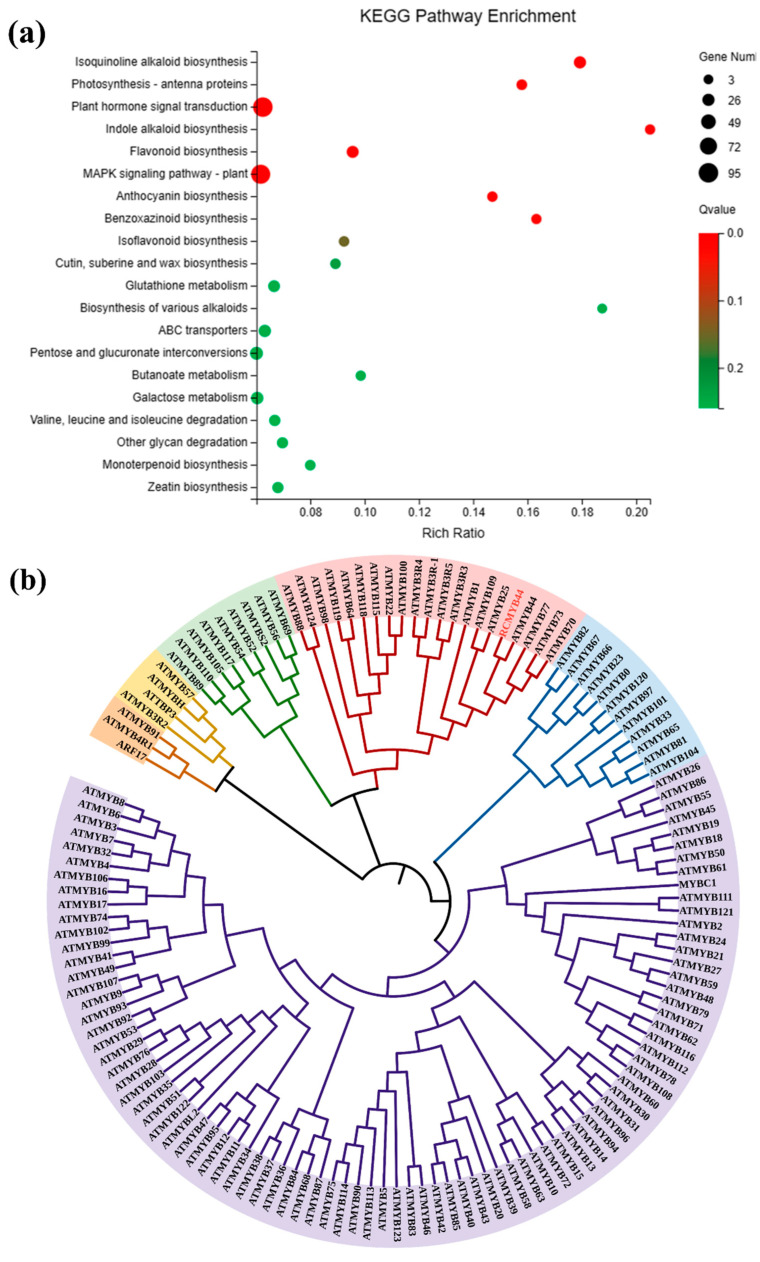

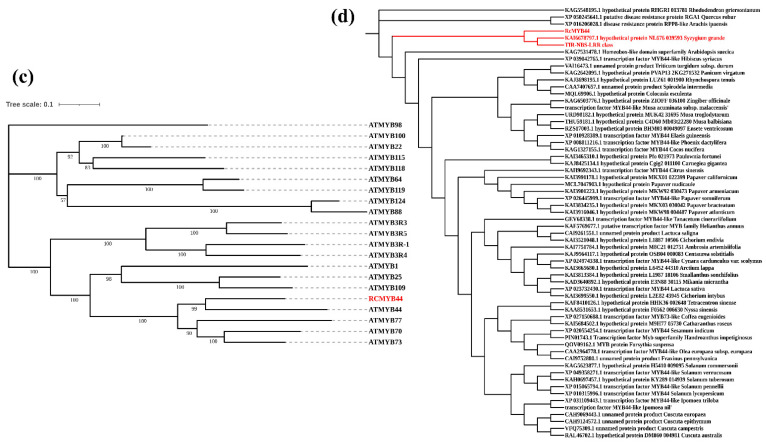
Functional prediction of *RcMYB44.* (**a**) KEGG-enriched pathways of DEGs in *R. chrysanthum* under UV-B radiation. The x-axis indicates the proportion of genes, and the y-axis indicates the pathway name. The color of the dots is related to the q-value; the size of the dots is proportional to the number of genes. (**b**) Construction of homologous evolutionary tree between *RcMYB44* and *Arabidopsis* MYB. (**c**) Sequence comparison with proteins from the same group as *RcMYB44*. (**d**) Functional analysis of *RcMYB44* homologous similar proteins in different species.

The enrichment analysis of the KEGG-based DEGs indicated that most of the KEGG pathways were highly enriched in *R. chrysanthum* under UV-B stress. The most enriched classifications were “plant hormone signal transduction” in *R. chrysanthum* under UV-B stress (95 DEGs) (Figure 2a). The identification and characterization of the stress-responsive TFs are essential to develop plants with enhanced tolerance. In our research, we found 2168 differentially expressed TFs. They comprised a variety of MYB, bHLH, NAC, and WRKY TFs associated with UV-B, and the diversity of their transcription profiles indicated that they play key roles in the UV-B stress response. The MYB (232 members) family had the largest number of differentially expressed TFs. The specific up-regulated or down-regulated TFs in *R. chrysanthum* may contribute positively to the UV-B tolerance. A total of four *RcMYB* transcription factors were found to be modified by acetylation in response to UV-B stress through joint analysis of the acetylated proteome and transcriptome. Of these, only *MYB44* has the full MYB structural domain. Through the proteomic analysis of the acetylation, acetylation was found to occur in *RcMYB44* (CL1734.Contig2-All) at the site 84, and *RcMYB44* was located in the nucleus (Table 1). 

To explore the function of *RcMYB44*, we compared it with the model organism *Arabidopsis* MYB and constructed a homology evolution tree. We divided the evolutionary tree into six groups, each labeled with a different color, with the pink group containing *RcMYB44* (Figure 2b). We analyzed this group exclusively and found that *RcMYB44* shares a common ancestor with *ATMYB44, ATMYB77, ATMYB73,* and *ATMYB70,* and *RcMYB44* is more homologous to *ATMYB44*, which is presumed to have the same function (Figure 2c). According to studies reported in the literature, during adversity stress, *ATMYB44* regulates the response of *Arabidopsis* to adversity stress through the salicylic acid, jasmonic acid, growth hormone, and abscisic acid signaling pathways [37,38,39]. To elucidate the mechanism of action of *RcMYB44* against UV-B stress we further analyzed proteins from other species homologous to *RcMYB44*. A blast homology comparison using NCBI was used to find proteins of other species with a similar homology, allowing us to further construct the evolutionary tree, which showed that only two proteins from two species might have the same function as *RcMYB44*, namely, *Medicago truncatula* and *Syzygium grande*, with the protein from *Medicago truncatula* being a disease-resistant protein and that from *Syzygium grande* being a hypothetical protein with an unknown function (Figure 2d).

### 3.3. Proteomics Reveals the Function of RcMYB44 in R. chrysanthum under UV-B Stress

**Figure 3:**
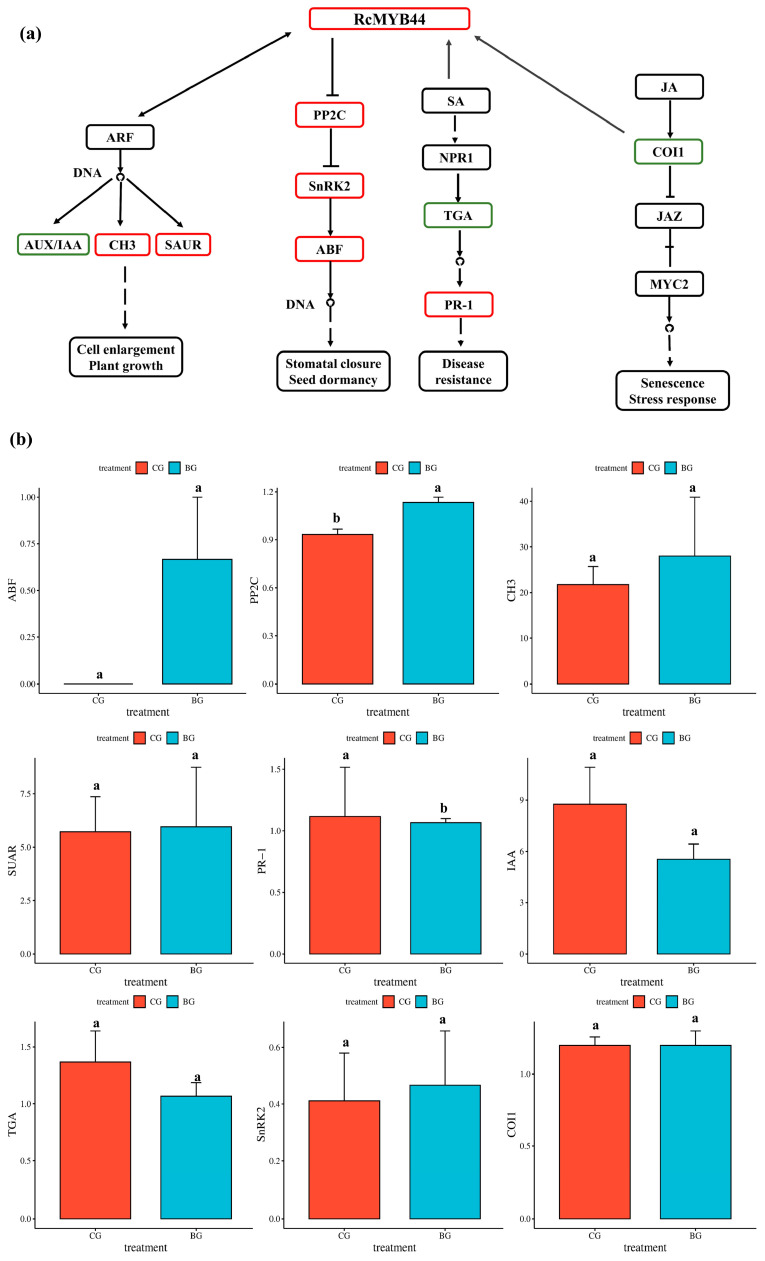
Protein expression (**a**,**b**) showing differential expression of *RcMYB44* involved in hormone signaling pathway proteins in response to UV-B treatment for 48 h in *R. chrysanthum*. (**a**) Analysis of proteins in hormone pathways involved in *RcMYB44*. Green indicates down-regulation of protein expression, red indicates up-regulation of protein expression. (**b**) Protein expression showing differential expression of proteins in hormone pathways. Small letters a and b indicate significant differences (*p* < 0.05).

To investigate the process of *RcMYB44* acting in the hormone signaling pathway, we constructed the relevant pathway in *R. chrysanthum*. Based on the construction of a previous homologous evolutionary tree, we suggest that the *R. chrysanthum RcMYB44* is involved in plant resistance to UV-B stress, mainly through growth hormone, abscisic acid, jasmonic acid, and salicylic acid pathway signaling (Figure 3). The proteomic analysis showed that *RcMYB44* expression increased under UV-B stress. During growth hormone signaling, the expression of the growth hormone AUX/IAA protein was down-regulated, while CH3 and SAUR proteins were up-regulated through the interaction of *RcMYB44* with ARF. In the abscisic acid signaling pathway, the expression of the negative regulator PP2C was up-regulated, and SnRK2 was activated and up-regulated to positively regulate ABF, which in turn affected the opening and closing of stomata to resist UV-B stress. In contrast, *RcMYB44* can participate in the jasmonate signaling pathway in response to UV-B stress. In the salicylic acid signaling pathway, *RcMYB44* mediates the expression of PR-1 to regulate plant resistance (Figure 3a). The expression of proteins of plant hormone signaling pathways, ABF, PP2C, TGA, PR-1, SnRK2, COI1, IAA, CH3, and SUAR, were studied in UV-B-stressed and PAR leaves. Under UV-B radiation, CH3, SAUR, PP2C, SnRK2, ABF, and PR-1 expression was enhanced in the UV-B-tolerant plants, and TGA, IAA, and COI1 expression decreased in the UV-B-tolerant plants (Figure 3b). PP2C expression was up-regulated by 19%, PR-1 1.08-fold, SnRK2 by 19.5%, SUAR by 4.1%, CH3 by 28.7%, TGA by 20.4%, and COI1 by 3.1%. Among them, the expression of ABF was detected only after UV-B treatment, suggesting that the ABA pathway plays an important role in the resistance of *R. chrysanthum* to UV-B stress.

### 3.4. Transcriptome Validation of RcMYB44 Function in R. chrysanthum under UV-B Stress

**Figure 4:**
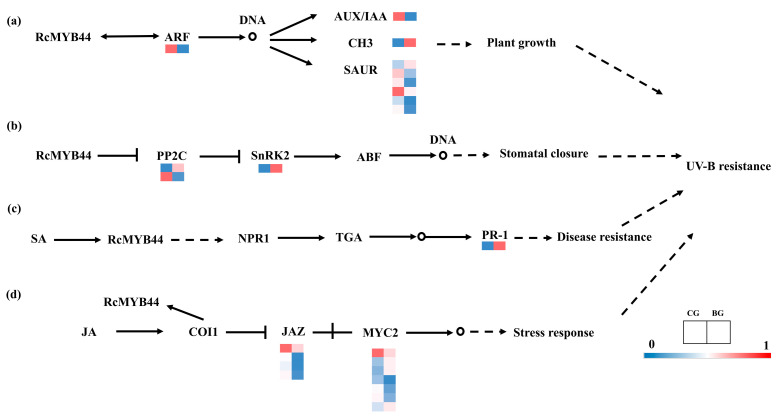
Transcriptome analysis of genes in hormone pathways involved in *RcMYB44*. (**a**) Auxin signal transduction pathway; (**b**) abscisic acid signal (ABA) transduction pathway; (**c**) salicylic acid (SA) signal transduction pathway; (**d**) jasmonic acid (JA) signal transduction pathway.

Transcriptomics was used to verify the expression of genes involved in the hormone transduction pathway. A total of 24 genes were annotated in the plant hormone signal transduction pathways (Figure 4). In the IAA, ABA, SA, and JA signaling pathways, 9, 3, 1, and 11 genes were found, respectively. In IAA, the expression of seven genes decreased under the 48 h UV-B treatment (Figure 4a) and the expression of two genes increased. This is in general agreement with the proteomic results. The expression of two genes (PP2C and SnRK2) in the ABA signaling pathway (Figure 4b) increased under the 48 h UV-B treatment, but the expression of one gene (PP2C) decreased. Induced by the UV-B treatment, the genes’ expressions in the SA signal transduction pathway (Figure 4c) changed in PR-1; the expressions of PR-1 increased in the 48 h UV-B treatment. In JA, the expression of nine genes decreased under the 48 h UV-B treatment (Figure 4d) and the expression of two genes increased. These results show that the ABA and SA signaling pathways were enhanced by the UV-B treatment, which is opposite to the case of the JA signaling pathway, and the IAA signaling pathway varied both up and down; together they constitute a system of resistance to UV-B stress in *R. chrysanthum*. The results show that the proteome and transcriptome are basically consistent, and some inconsistencies may be related to post-translational modifications of proteins.

### 3.5. Three-Dimensional Structure Construction and Noncovalent Interaction Analysis of RcMYB44 from R. chrysanthum

**Figure 5:**
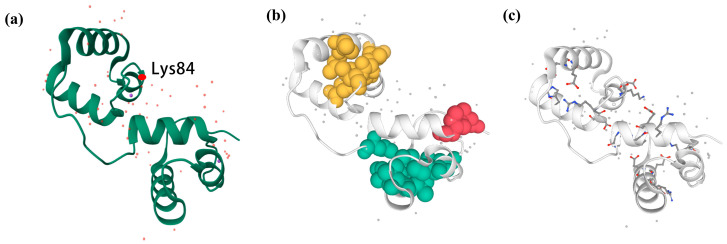
Three-dimensional structure of *RcMYB44*. (**a**) Visualization of three-dimensional structure of *RcMYB44* and labeling of acetylation sites. (**b**) Visualization of hydrophobic cluster of *RcMYB44*. (**c**) Visualization of *RCMYB44* salt bridge.

Based on the above information, we constructed the three-dimensional structure of *RcMYB44* and labeled the acetylated site (Figure 5a). Proteins fold into their natural structures driven by a variety of non-covalent interactions; thus, to understand protein characterization and function at the molecular level, interactions must be described. The visualization of these structures using ProteinTools determined that *RcMYB44* contained three hydrophobic clusters, with the largest hydrophobic cluster area being 770.6^2^ (Figure 5b, Table 2). The analysis of the hydrophobic clusters showed that cluster 0 had a total area of 110.4^2^, this cluster contained one residue, an area of 55.2^2^ per residue, and two interactions between residues. Cluster 1 had a total area of 589.5^2^, this cluster contained six residues, an area of 45.3^2^ per residue, and 13 interactions between residues. Cluster 2 had a total area of 770.6^2^, this cluster contained seven residues, an area of 45.3^2^ per residue, and 17 interactions between residues. The salt-bridge networks in the proteins were found and the charge separation parameters calculated. Analysis of the results showed that there are eight salt bridges in *RcMYB44* (Figure 5c). By calculation, it was found that the FCR (fraction of charged residues) was 0.34, and the K (kappa value) was 0.17.

## 4. Discussion

As a result of the degradation of the stratospheric ozone layer, the quantity of UV-B radiation that reaches both the Earth and plant surfaces has increased [40]. Therefore, we need to better understand the molecular mechanisms of plant resistance to UV-B radiation. However, to date only minor attention has been paid to the integration of transcriptomic and acetylated proteomic data to analyze how plants respond to UV-B. In our research, we obtained MYB TFs that underwent acetylation modifications through a combined acetylated proteome and transcriptome analysis. By constructing a homologous evolutionary tree, we believe that *RcMYB44* has the same function as the homologous *ATMYB44*. *RcMYB44* can resist UV-B stress by mediating the growth hormone, abscisic acid, jasmonic acid, and salicylic acid signaling pathways. We also constructed the hydrophobic and salt-bridge structures of *RcMYB44*, which is an important and unique part of this study. 

On the basis of the data of the transcriptome and acetylated proteome obtained, the mRNA and proteins of *R. chrysanthum* leaves were significantly different from those exposed to UV-B. In total, 2348 DEGs and 807 DEPs were identified. In *R. chrysanthum*, there are great differences between the DEGs and DEPs. This is also supported by many earlier transcriptomic studies with similar factors [41,42]. For example, 1210 DEGs have been found in loquat under cold stress, but only 300 DEPs [42]. There were two major causes for the low correlation between the transcriptome and the proteome. On the one hand, even though similar plant material and stress treatments were utilized, the two kinds of omics analyses were not carried out simultaneously, which had a certain impact on the relationship. Furthermore, post-transcriptional modification and turnover of the proteins also affected the levels of proteins.

Histone acetylation has defined mechanisms of action in all kinds of biological processes [43,44]. However, the mechanisms of action of non-histone acetylation modifications are less well known. It was reported that acetylation modification of key enzymes in primary metabolism plays an important role in the release of poplar (*Populus tremula × Populus alba*) buds from dormancy [32]. Elevated acetylation levels of *PuMYB110a* in pear promoted anthocyanin accumulation in the fruit [45]. Increasing the level of acetylation of the *PuWRKY31* promoter in pear fruit is a mechanism for increasing the sugar content [46]. Based on the results of other studies, we found a large number of acetylation sites in proteins of *R. chrysanthum*, strongly and convincingly suggesting the significance of Kac modification for UV-B resistance in plants [47]. In addition, in *R. chrysanthum*, we note that several classes of transcription factors undergo lysine acetylation modifications (Table 1); the expression of these TFs was significantly altered under UV-B stress, and we hypothesize that acetylation modifications may regulate the activity of these transcription factors. This study is the first to report on the acetylation modification of transcription factors in *R. chrysanthum*.

In plants, the MYB TF family is a large family with diverse and intricate functions [48]. It has been reported that MYB transcription factors regulate plant responses to stress. For example, *GmMYB81* induces plant seed germination under abiotic stresses, for example, drought stress, low temperature stress, and salt stress [49]. Under UV-B irradiation, the UV-B photoreceptor *UVR8* of *Arabidopsis* interacts with different MYB transcription factors to control cotyledon unfolding and lateral root growth in the cotyledons and roots of the plant, respectively [50]. The promoter of *AtMYB4* may be involved in the regulation of its *Arabidopsis* expression under UV-B light [51]. The overexpression of *AaMYB1* in *Artemisia annua* resulted in increased flavonoid biosynthesis to resist UV-B stress [52]. In this work, *RcMYB44* could participate in the growth hormone, abscisic acid, jasmonic acid, and salicylic acid signaling pathways to improve UV-B resistance in *R. chrysanthum* (Figure 3). Under the condition of UV-B radiation, our research identified 232 MYB family members in *R. chrysanthum*. *RcMYB44* undergoes acetylation modification and its expression is up-regulated, and homology analysis showed that *RcMYB44* has high homology with *ATMYB44* in *Arabidopsis*. Therefore, we believe that *RcMYB44* has the same function as the homologous *Arabidopsis ATMYB44*; it is tolerant to adversity stresses during plant growth. Consequently, it will be necessary and meaningful to investigate how *RcMYB44* enhances plant resistance to UV-B stress through intrinsic mechanisms.

**Figure 6:**
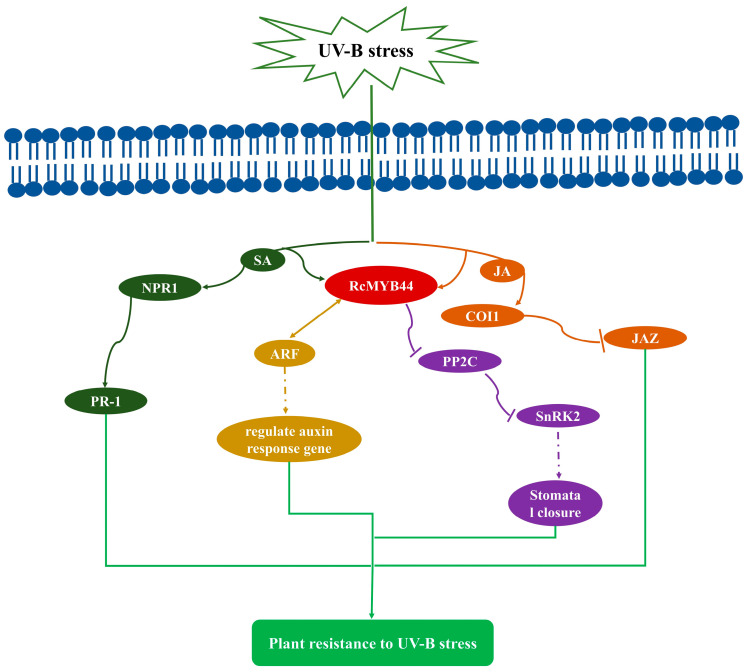
*RcMYB44* is involved in hormone signaling pathways in *R. chrysanthum* under UV-B stress. *R. chrysanthum RcMYB44* resists UV-B stress by participating in growth hormone, abscisic acid (ABA), jasmonic acid (JA) and salicylic acid (SA) signaling pathways.

We believe that UV-B causes damage to plants, but plants resist UV-B stress by regulating their own molecular mechanisms. In *R. chrysanthum*, *RcMYB44* can mediate four pathways to resist UV-B stress, namely, interacting with ARF to regulate the expression of growth hormone genes; acting on the ABA signaling pathway to regulate the opening and closing of the stomata to resist UV-B stress; and acting on the jasmonic acid and salicylic acid signaling pathways to resist UV-B stress (Figure 6). These will be the focus of our future research.

## 5. Conclusions

In conclusion, a total of four acetylated MYB transcription factors were identified in this study by combining the transcriptome and acetylated proteome analysis. Only *RcMYB44* has a complete MYB domain. To investigate the function of *RcMYB44*, we constructed a homologous evolutionary tree between *RcMYB44* and *Arabidopsis* MYBs and identified a functionally similar homologous protein *ATMYB44*. The function of *RcMYB44* can be obtained through the function of *ATMYB44*. *RcMYB44* undergoes acetylation modification and its expression level is up-regulated. In the process of auxin signal transduction, *RcMYB44* interacts with ARF to regulate the expression of downstream auxin genes. In the process of abscisic acid signal transduction, *RcMYB44* acts on this pathway, regulating stomata to resist UV-B radiation. In the signal transduction pathway of salicylic acid, *RcMYB44* mediates the expression of PR-1 to regulate plant resistance. Based on the function of *ATMYB44*, it can be concluded that the *RcMYB44* in *R. chrysanthum* mediates the resistance to UV-B stress in the growth hormone, abscisic acid, jasmonic acid, and salicylic acid signaling pathways.

## Figures and Tables

**Table 1:** Information of *RcMYB* acetylation modification in *R. chrysanthum*.

Protein Accession	Position	Amino Acid	Gene Name	Subcellular Localization
CL1734.Contig2_All	84	K	*MYB44*	nucleus
Unigene7559_All	151	K	*MYB1*	chloroplast
Unigene12045_All	172	K	*-*	chloroplast
Unigene11720_All	249	K	*ADA2*	nucleus

**Table 2:** Information about hydrophobic cluster of *RcMYB44*.

Cluster ID	Area	No. Contacts	Contacts/Residue	Area/Residue
0	110.4	2	1.0	55.2
1	589.5	13	1.9	45.3
2	770.6	17	2.4	45.3

## Data Availability

The datasets generated during or analyzed during the current study are available from the corresponding author on reasonable request.

## Supplementary Materials

The following supporting information can be downloaded at: https://www.mdpi.com/article/10.3390/genes14112022/s1.
